# Impact of maternal dietary counseling in the first year of life on DNA methylation in a cohort of children

**DOI:** 10.1590/1678-4685-GMB-2020-0330

**Published:** 2021-12-03

**Authors:** Janaína Kehl de Castilhos, Paula Dal Bó Campagnolo, Silvana de Almeida, Márcia Regina Vitolo, Vanessa Suñé Mattevi

**Affiliations:** 1 Universidade Federal de Ciências da Saúde de Porto Alegre, Porto Alegre, RS, Brazil.; 2 Universidade do Vale do Rio dos Sinos, São Leopoldo, RS, Brazil.

**Keywords:** Epigenetics, DNA methylation, dietary intervention, metabolic diseases

## Abstract

Epigenetic modifications established during prenatal and early life, including DNA methylation, have been suggested as potential mediators of the interaction between environmental exposures during the perinatal period and adult metabolic health adverse outcomes, especially cardiometabolic complications and overweight. The effect of a dietary intervention in the first year of life on global methylation levels in leukocyte samples from a cohort of children born between 2001 and 2002 in southern Brazil was examined. Overall methylation measurements were performed using enzyme-linked immunosorbent assays on DNA samples from 237 children at 4 years old. Mean methylation values were higher in the intervention group (mean: 2.20 ± 1.31%) than in the control group (mean: 1.65 ± 1.11%; *P* = 0.001). It was observed that nutritional counseling in the first year increased breastfeeding duration and stimulated the development of healthier eating habits. Therefore, these factors might have contributed to increase global DNA methylation. The findings of the present study reinforce the notion that performing nutritional interventions in the early stages of life is important and provide further evidence of the interaction between the environment and epigenetic traits.

## Introduction

In recent decades, the world has witnessed a dramatic increase in the incidence of childhood overweight, obesity, hypertension, dyslipidemia, insulin resistance, and diabetes, which have been considered serious public health problems (Garcia-Cardona et al., 2014; Dave et al., 2015). An emerging hypothesis to explain this increase is that a higher exposure to an “obesogenic” environment in early life may contribute to this epidemic in childhood. Such metabolic alterations have a multifactorial etiology that involves complex interactions among genetic background, hormones, and different environmental factors (McPherson, 2007; Herrera and Lindgren, 2010). Animal models and human data have shown that epigenetic modifications can underlie these interactions and their impact on metabolic changes (Houde et al., 2013; Moleres et al., 2013; Waterland, 2014), as exemplified by the studies on the Dutch famine of 1944-45 (Heijmans et al., 2008).

DNA methylation is one of the most widely investigated epigenetic marks. It is essential for normal development and contributes to gene regulation. DNA methylation is usually associated with changes in gene transcription; low levels of methylation, generally in the promoter regions of genes, can lead to increased protein expression (Houde et al., 2015; Samblas et al., 2019).

One of the most important moments to prevent diseases in adulthood is early life. Meta-analyses have shown that breastfeeding is a protective factor for obesity, and global DNA methylation patterns are known to be altered by early nutrition (Yan et al., 2014; Hartwig et al., 2017). Understanding how maternal diet and early nutrition modulate epigenetic marks may be relevant in reducing the risk of future obesity and related metabolic disturbances (Samblas et al., 2019). Recent ideas have emphasized that the “first 1000 days” of life constitute the most important window in the programming of future health and disease; they also offer hope that an intervention in early life could help prevent these disorders (Yajnik, 2014).

Intervention studies are essential to elucidate the impact of the early life environment on epigenetic markers. Longitudinal cohort studies are costly to perform and sustain because they should include extensive, prospectively collected data and biological samples at multiple time points across the life course. However, these studies can provide an opportunity to increase the understanding of the dynamic nature of epigenetic patterns and how changes occur in response to environmental, lifestyle, and behavioral factors (Ng et al., 2012). Intervention studies regarding the relationship between epigenetic modifications, early life exposures, and metabolic disease development, such as overweight, obesity, hypertension, dyslipidemia, and insulin resistance, in adult life in humans are still scarce.

In 2001-2002, the present group performed a randomized trial aiming to assess the impact of dietary counseling given to mothers during the first year of infants’ lives on food consumption, nutritional status, and lipid profiles of 500 children from a low-income population setting from the South of Brazil. These children have been under follow-up since birth until 12 years of age. These studies have shown that such an intervention positively influences breastfeeding and reduces the occurrence of morbidities, such as diarrhea, respiratory problems, dental caries, and medication use at one year of age (Vitolo et al., 2005). Similarly, this dietary counseling during the first year of life was found to improve the healthy eating index measured in these children at preschool age (Vitolo *et al.*, 2010). Genetic variants associated with overweight and an increase in body mass index at 4 years of age were also found by our group in this study population (da Silva *et al.*, 2013; Zandona et al., 2013; Zandona *et al.*, 2017).

While the effect of the fetal and early childhood environments on health outcomes in adult life has been widely discussed, the empirical assessment of this hypothesis remains a challenge (Lumey et al., 2015). Based on this information and knowing that the early stages of life are considered critical windows for genetic and epigenetic modifications and for the establishment of habits that influence lifelong health patterns, we hypothesized that the effect of dietary counseling could be due to changes in methylation levels in this cohort. Therefore, the present study evaluated DNA samples collected at 4 years old from children enrolled in the previously mentioned healthy feeding-promoting study in order to evaluate the effect of that intervention on global DNA methylation levels.

## Material and Methods

### Subjects

**Figure 1 -:**
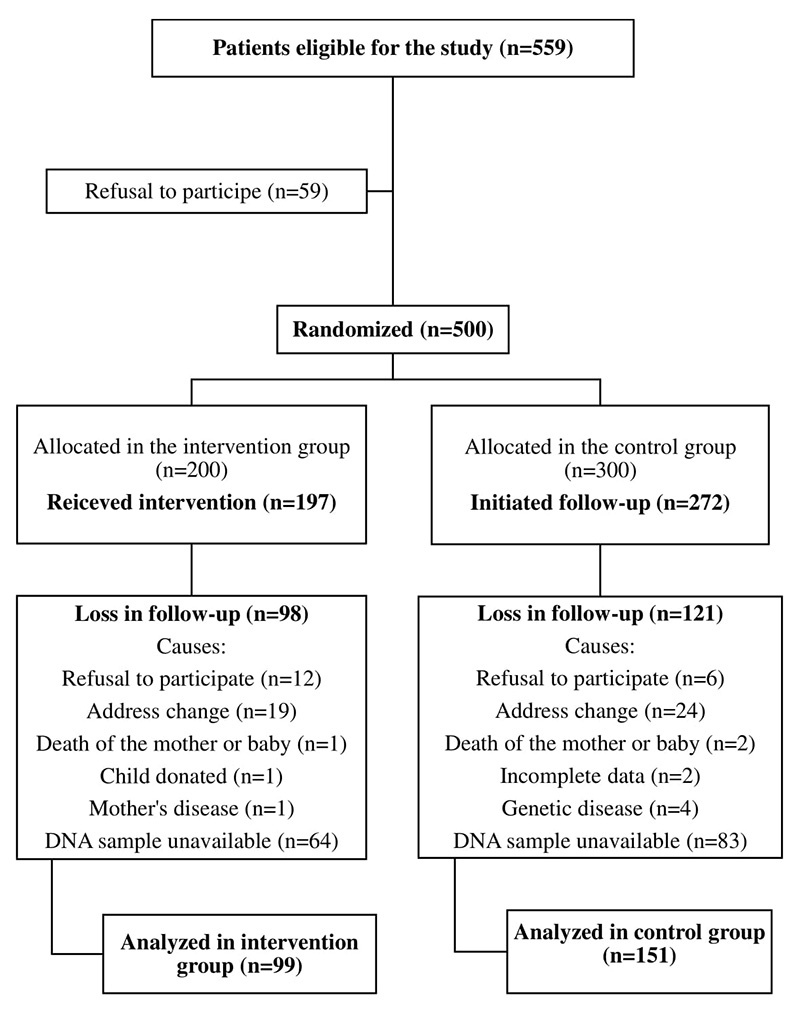
Study overview.

This research is part of a cohort study that included 500 children born between 2001 and 2002 in a public-funded hospital in southern Brazil. Inclusion criteria were healthy, singleton, full-term (> 37 weeks), and normal birth weight (> 2500 g). The exclusion criteria were HIV-positive mothers, infants with congenital malformations, infants who were admitted to neonatal intensive care units, and individuals with breastfeeding impediments. The children were randomly assigned to the control and intervention groups at birth. The intervention group followed a diet based on the “Ten Steps to Healthy Feeding for Brazilian children from birth to two years of age”, as advised by the Brazilian Ministry of Health, during the first year of life (Vitolo et al., 2005). For children in the intervention group, ten home visits were performed: the first at day 10 after birth, then monthly up to 6 months of life, and then at 8, 10, and 12 months. Home visits for children in the control group occurred at 6 and 12 months of life. During home visits, anthropometric, dietary, socioeconomic, demographic, and health data were collected. These data and biological samples for biochemical evaluation were collected from all children at the ages of 1, 4, 8, and 12 years. Data collected until four years of age were analyzed. Participant recruitment and follow-up data are summarized in Figure 1. More detailed information on randomization and data collection in this cohort trial is available in the study by Costa et al. (2017). The study was approved by the Research Ethics Committees of the institutions involved (number 18426813.4.0000.5344), and all mothers gave their informed consent to participate in the study.

### Nutritional analysis

One 24-hour dietary recall was collected during home visits for each child at 1 year of age, and two 24-hour dietary recalls were collected on two nonconsecutive days at 4 years of age; the mean values were used in the analyses.

The Healthy Eating Index (HEI) is an indicator used to measure diet quality. It evaluates different aspects of healthy nutrition through 10 components, based on the individual’s nutritional needs. For this study, the consumption of 6700 kJ (1600 kcal) was recommended, a criterion adopted for children between three and four years. Further information on the calculation of this indicator in this sample can be found in the study by Vitolo et al. (2010). The composite HEI score may range from a minimum of 0 to a maximum of 100. In this study, a total score higher than 80 was considered “good,” scores between 51 and 80 indicated “needs improvement,” whereas scores below 51 were considered “poor.”

Foods with a sugar composition of 50% or more in 100 g (e.g., candies, soft drinks, sugar, and honey) were considered high sugar density foods, while those that had 30% or more fat content in 100 g (e.g., salty snacks, filled cookies, and chocolate) were considered high lipid density foods.

### Global methylation analysis

DNA samples were obtained from peripheral leukocytes using a standard salting-out technique from biological samples collected at 4 years of age. DNA methylation was quantified by enzyme-linked immunosorbent assay, and results were expressed in terms of percent methylation (percentage of deoxy-methyl cytosine; % dmC). The MethylFlash™ Methylated DNA Quantification Kit (Colorimetric, Base Catalog # P-1034; Epigentek Group INC., Farmindgale, NY, USA) was used to analyze global methylation. Experiments were performed in accordance with the manufacturer´s instructions and using appropriate controls; 80 ng per sample were used as DNA input.

### Statistical analysis

The homogeneity of the samples regarding categorical anthropometric, dietary, demographic, and socioeconomic variables distribution among the control and intervention groups subsequent to randomization was verified using chi-squared tests.

DNA methylation and triglyceride levels were asymmetrically distributed and logarithmically transformed prior to statistical analyses. Untransformed values are presented in the tables to facilitate interpretation. The other continuous variables evaluated presented a normal distribution. Continuous variables were compared between groups using independent samples *t*-tests.

Univariate linear regressions were used to assess the correlations between continuous variables. Multiple linear regressions were used to assess the effect of the intervention and duration of exclusive breastfeeding on DNA methylation levels. The models were adjusted for sex and age at 3 to 4 year evaluations. Data are expressed as unstandardized regression coefficients (B), 95% confidence intervals (CI), regression coefficients (r), and *P*-values.

Statistical analyses were performed using SPSS version 22.0 for Windows (IBM, Armonk, NY), and differences were considered significant when *P* < 0.05.

## Results

Among the 500 children that were initially allocated to either the control (n = 300) or intervention (n = 200) groups back in 2001-2002, only 354 were found for 4th-year interviews in 2005-2006, and complete data were obtained for only 345 of them (Costa et al., 2017). For this study, global DNA methylation analyses were performed on the 250 DNA samples available from some of these children (3- to 4-years old); 151 samples belonged to the control group and 99 to the intervention group (Figure 1). After evaluating the distribution of the DNA methylation levels (% dmC) in the samples, 13 of them were considered as outliers because their methylation levels were outside the first or third quartile range (1.5× interquartile interval). These samples were excluded from further analysis.

The descriptive characteristics of the children analyzed and categorized in the intervention and control groups are presented in Table 1. Among the 237 children analyzed in the study, 132 (55.69%) were boys. The proportion of boys and girls did not differ between groups (Table 1). The table also shows that mothers from both groups were similar in terms of gestational weight gain and smoking habits during gestation. The only significantly different parameter between groups was the exclusive breastfeeding duration. The proportion of infants in the intervention group that received breast milk exclusively for 4 months or more was 41.8%, in contrast to 25.9% in the control group (*P* = 0.011). The total breastfeeding duration over 12 months was 50.5% in the intervention group and 42.0% in the control group, but this difference was not statistically significant.

**Table 1 -:** Characteristics of children according to the study groups.

		Intervention		Control		*P*-values^a^
		n	%	n	%	
Sex	Boys	54	58.7	78	53.8	0.459
	Girls	38	41.3	67	46.2	
Total breastfeeding duration	<12 months	45	49.5	83	58.0	0.198
	≥12 months	46	50.5	60	42.0	
Exclusive breastfeeding duration	<4 months	53	58.2	106	74.1	0.011
	≥4 months	38	41.8	37	25.9	
Gestational weight gain	Low	33	40.2	40	30.1	0.214
	Adequate	27	32.9	44	33.1	
	Excessive	22	26.8	49	36.8	
Maternal smoking during gestation	Yes	8	9.3	23	16.8	0.116
	No	78	90.7	114	83.2	

^a^ *P-*values from chi-squared tests.

Regarding health status, 12.7% of the children were overweight and 3.6% of the children were obese in the total sample at preschool age (4 years old). As depicted in Table 2, the proportion of overweight and obese children did not differ between the intervention and control groups. Lipid profiles (total cholesterol, high-density lipoproteins, and triglycerides) were mostly within the recommended ranges and were not different between the groups. The proportion of children with a good diet as classified by the HEI was higher in the intervention group, and the frequency of children with a poor diet was higher in the control group. None of these health status variables was associated with DNA methylation levels (Table S1).

**Figure 2 -:**
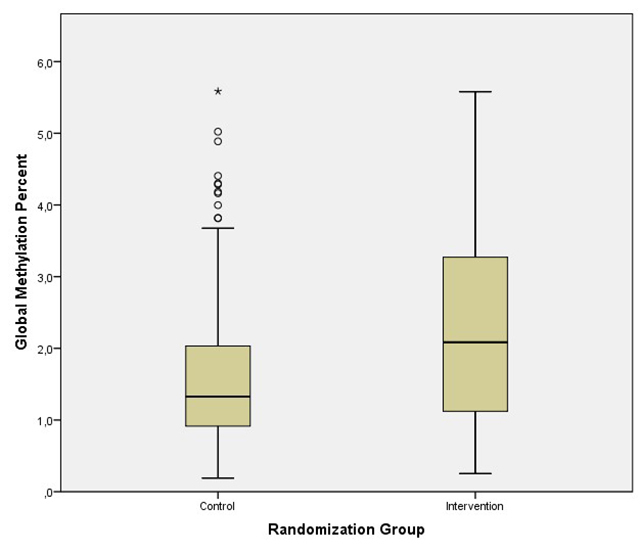
Boxplots showing a comparison between the DNA methylation levels of the intervention and control groups. Data are presented as medians and range of variation. Boxes represent the intervals between the first and third quartiles. Comparison was made through a *t*-test for independent samples with ln-transformed values, *P* < 0.001.

A highly significant difference in mean global methylation values was found between control and intervention groups (intervention group mean: 2.20 ± 1.31%; control group mean: 1.65 ± 1.11%; independent samples *t*-test = 3.24; *P* = 0.001; Figure 2).

**Table 2 -:** Health status of children at 4 years according to the studied groups.

		Intervention		Control		*P*-values
		n	Mean (±SD) or %	n	Mean (±SD) or %	
Age (years)		89	3.97 (0.22)	143	3.97 (0.21)	0.783^a^
Weight status	Normal	68	80.0	116	85.9	0.320^b^
	Overweight	12	14.1	16	11.9	
	Obese	5	5.9	3	2.2	
High cholesterol (≥ 170 mg/dl)		4	4.9	11	8.0	0.581^b^
Low HDL cholesterol (≤ 35 mg/dl)		13	15.9	29	21.0	0.445^c^
High Triglycerides (≥ 100 mg/dl)		5	6.1	4	2.9	0.248^b^
HEI	Poor	4	4.7	20	14.3	0.036^c^
	Needs improvement	69	81.2	109	77.9	
	Good	12	14.1	11	7.9	

^a^ independent samples *t*-test; ^b^ Fisher’s exact test; ^c^ chi-squared test; *P*-value for triglycerides was calculated from the ln-transformed variable; HDL, high-density lipoprotein; HEI, healthy eating index.

The influence of other environmental factors to which children were exposed and that were considered as possibly relevant for the study outcome (% dmC) was also evaluated (Tables 3 and 4). Table 3 shows the results from the analysis done considering several pre-gestational (weight of the mother), gestational (smoking, mother weight gain, sex of the child), and postnatal (type of delivery, exclusive breastfeeding up to 4 months, total breastfeeding time, group in which the child was allocated, consumption of high lipid and high sugar density foods in the first year of life) variables, as well as the living environment (smoking-free or smoking by the mother and/or someone else in the house) at 4 years of age. None of these variables were associated with % dmC. For the continuous variables available (pre-gestational BMI, total breastfeeding duration, exclusive breastfeeding duration, amount of ingested folate and B12 vitamin in the first year of life, and healthy eating index in the fourth year; Table 4), univariate regressions with % dmC were evaluated, but none of them was significantly associated with this outcome.

A multivariate regression model adjusting for the covariables children’s age, sex, and duration of exclusive breastfeeding, revealed only a significant association between the nutritional counseling intervention performed and the % dmC, as shown in Table 4. A second model evaluating only the effect of the intervention, adjusting for age and sex, presented in the same table, showed that the intervention increased the DNA methylation levels by 0.273%.

**Table 3 -:** Comparison of methylation levels according to the environmental factors to which children were exposed.

		n	Mean (% dmC)	SD (% dmC)	*P*-values ^*a*^
Pre-gestational weight	Eutrophic	143	1.87	1.28	0.742
	Overweight	56	1.84	1.05	
	Obese	21	1.88	1.15	
Gestational smoking	Yes	31	1.86	1.26	0.686
	No	192	1.88	1.22	
Gestational weight gain	< 9.0 kg	69	2.11	1.30	0.191
	9.1 - 15.9 kg	92	1.80	1.18	
	>16.0 kg	63	1.66	1.28	
Sex of the child	Boys	132	1.89	1.23	0.416
	Girls	105	1.83	1.21	
Type of delivery	Normal	123	1.79	1.23	0.148
	Caesarean	85	1.94	1.16	
High lipid density foods consumption (at 1 year)	Yes	107	1.73	1.19	0.065
	No	127	1.95	1.20	
High sugar density foods consumption (at 1 year)	Yes	62	2.02	1.38	0.628
	No	172	1.78	1.12	
Smoker mother (at 4 years)	Yes	49	1.92	1.31	0.976
	No	173	1.83	1.18	
Other smoker in the household (at 4 years)	Yes	81	1.71	1.17	0.109
	No	150	1.93	1.21	

% dmC, percentage of deoxy-methyl cytosine; SD, standard deviation.

^a^ Independent samples *t*-test with *ln*-transformed methylation levels

**Table 4 -:** Linear regressions between environmental factors and global DNA methylation in children

Univariate models					
	B	95% CI	r	*P*	
Pre-gestational BMI	0.013	-0.006, 0.033	0.089	0.188	
Exclusive breastfeeding duration	0.027	-0.015, 0.069	0.083	0.208	
Total breastfeeding duration	0.002	-0.007, 0.011	0.049	0.613	
Folate ingestion amount (at 1 year)	-0.034	-0.002, 0.001	0.034	0.617	
B12 vitamin ingestion amount (at 1 year)	0.002	-0.010, 0.013	0.018	0.793	
HEI at 4 years of life	-0.001	-0.009, 0.008	0.012	0.855	
Intervention	0.305	0.119, 0.491	0.206	0.001	
Multivariate models					
	B	95% CI	*P*	r	*P* ^*a*^
Model I					
Exclusive breastfeeding duration	0.014	-0.029, 0.057	0.530	0.188	0.090
Age (years)	-0.114	-0.548, 0.314	0.605		
Gender (boys)	0.015	-0.170, 0.199	0.876		
Intervention	0.244	0.053, 0.436	0.013		
Model II					
Age (years)	-0.144	-0.569, 0.280	0.504	0.195	0.031
Gender (boys)	0.022	-0.162, 0.207	0.811		
Intervention	0.273	0.087, 0.460	0.004		

B, unstandardized regression coefficient; CI, confidence interval; r, regression coefficient; ^a^ *P* value of ANOVA for the models including all variables. BMI, body mass index; HEI, healthy eating index. Global methylation levels were *ln*-transformed.

## Discussion

In this study, our main goal was to verify whether nutritional counseling performed in the first year of life had an impact on global DNA methylation in children. Furthermore, we aimed to understand how some environmental factors faced in the early stages of life, such as the nutritional status of the mother during pregnancy, smoking, and breastfeeding, as well as sex, were related to DNA methylation.

We found a highly significant difference in mean methylation levels between the intervention and control groups, where the intervention apparently increased global DNA methylation. A previous work by some of us (Vitolo et al., 2008) showed that both groups were similar regarding socio-economic variables, such as family income, maternal schooling and age, and family structure at the time of the child’s birth. All families had low incomes and social conditions. Our data also indicated that the groups were similar in age, sex, and exposure to several environmental factors during the intrauterine period, such as cigarette smoke, mother’s weight gain, and type of delivery. In addition, these data showed the effectiveness of the randomization of the mothers between the control and intervention groups, confirming that the intervention stimulated the administration of breast milk as the only nutritional source for a longer period and resulted in a better index of healthy eating for preschool children.

The secondary goal of the present study was to evaluate which environmental factors could exert a greater interaction with the genetic material of children in the postnatal period through DNA methylation. The consumption of foods rich in sugar and lipids, the intake of folate and vitamin B12 in the first year of life, and the intake of breast milk were not associated with the overall methylation levels of the genome. We also found no association between DNA methylation levels and exposure to cigarette smoke and healthy eating up to 4 years of age.

Many studies have demonstrated that the nutritional status of the mother and metabolic dysregulation during gestation are crucial factors for the development and health of the next generation (Bouchard et al., 2010; Kulkarni et al., 2011; Bouchard, 2013; Soubry et al., 2015). The Developmental Origins of Health and Disease (DOHaD) theory describes a strong association between life events (pre- and postnatal) and biological/epigenetic responses that define the risk of developing diseases. These studies have confirmed that the periods of greater epigenetic plasticity are the prenatal, neonatal, and pubertal stages during which epigenetic markers are associated with disease risk and can be modified through lifestyle changes (Dolinoy et al., 2007; Agarwal et al., 2018). Our findings indicate that the intervention performed in the first year of life was highly significantly associated with global DNA methylation levels. Based on these premises, we attempted to evaluate the possible influence of different isolated pre- and perinatal factors on DNA methylation levels of the children of the studied cohort at 4 years of age. However, none of the analyzed factors was associated with DNA methylation. 

The “Ten Steps to Healthy Feeding for Brazilian children from birth to two years of age” is a guideline developed by the Brazilian Ministry of Health that focuses on the increase in the duration of breastfeeding together with the appropriate introduction of healthy complementary foods at the correct age. It also addresses the importance of avoiding non-healthy foods, such as those rich in sugar, fat, and salt. Although at the present moment we were not able to observe differences in the health status of children that were subjected to the intervention, a previous publication by our group showed that the intervention was associated with a lower proportion of children with diarrhea, respiratory problems, use of medication, and dental caries in the 12-16 month- old period (Vitolo et al., 2005). Longitudinal studies and follow-up of our cohort may be important to address the long-term effects of this intervention. In previous studies with the same cohort of children, it was observed that those with excessive weight gain in early life had an increased susceptibility to insulin resistance at 8 years of age (Costa et al., 2017). In addition, it was observed that nutritional counseling in the first year of life improved early feeding practices and improved the lipid profile of these children at 8 years of age (Louzada et al., 2012). In this context, we suggest that these better practices as a whole may affect DNA methylation levels at 4 years and speculate that they could be protective in the long term for the development of complex diseases such as obesity. Thus, our work reinforces the evidence that the mechanism behind the effects of good nutrition to promote a healthy life involves interaction with our genome through epigenetic changes.

The present study is innovative in evaluating DNA methylation levels in a human population that underwent dietary intervention. The literature regarding the epigenetic effects of nutritional interventions in humans is scarce, but some studies regarding the effect of breastfeeding have been found. In 2017, Hartwig *et al.*, in a systematic review, found five studies conducted in humans on the effects of breastfeeding on DNA methylation. These investigations were highly heterogeneous, but in all the papers analyzed by the authors associations between breastfeeding and DNA methylation were shown. In an article published in *Nutrients* in 2014, Verduci *et al* *.* hypothesized several ways in which breast milk could have a beneficial effect on children’s health and decrease the development of complex diseases such as obesity in adulthood. An involvement of epigenetic mechanisms, especially DNA methylation, in the beneficial impact of breastfeeding is possible. However, it is difficult to clearly state how breastfeeding modifies DNA methylation levels. Hypothetically, nutrient supply might interfere with one-carbon metabolism, regulating the availability of methyl groups for biological methylation reactions (Verduci *et al.*, 2014; Friso et al., 2017).

The main limitation found in the reviewed articles is the lack of studies on this topic, which makes the mechanisms of interaction between nutrition and DNA methylation still poorly understood. Moreover, this lack of information may hinder the evaluation of potentially confounding variables, which was also performed in our study (Obermann-Borst et al., 2013; Hartwig et al., 2017). On the other hand, our longitudinal study may increase our capacity to recognize the real impact of environmental factors on epigenetic mechanisms and their influence on the pathophysiology of complex diseases in adult life (Ng et al., 2012). We are aware that the global methylation analysis performed here was based on a poor resolution approach compared with genome-wide profiling methods. However, our findings provide preliminary evidence that should be used to support future studies with more robust locus-specific analyses or genome-wide DNA methylation profiling.

In summary, we report the association between early life nutritional intervention and global DNA methylation levels in young children. Although far from conclusive, our findings strengthen the importance of performing nutritional interventions in the early stages of life and reinforce the evidence of a mechanism for these effects through DNA methylation. Further investigations are needed to understand the complex interactions between environmental factors such as nutritional patterns, and epigenetic mechanisms, in order to improve therapeutic strategies for preventing most adult chronic diseases.

## Supplementary material

The following online material is available for this article:

Table S1 - Univariate linear regressions between health status variables at 4 years and global DNA methylation in children.
