# Supplementary material for: Impact of maternal dietary counseling in the first year of life on DNA methylation in a cohort of children
Source: Genet Mol Biol. 2021 Dec 3;44(4):e20200330. doi: 10.1590/1678-4685-GMB-2020-0330 (PMC8670011; doi:10.1590/1678-4685-GMB-2020-0330)
Supplement: Table S1 - [file 1415-4757-GMB-44-4-e20200330-s1.pdf]

Genetics and Molecular Biology
https://doi.org/10.1590/1678-4685-GMB-2020-0330

Supplementary Material to "Impact of maternal dietary counselling in the first year of life on DNA methylation in a cohort of children"

Table S1 – Univariate linear regressions between health status variables at 4 years and global DNA methylation in children.

	B	95% CI	*P*
BMI Z-score	0.044	-0.036, 0.124	0.277
Fasting glucose (mg/dL)	-0.001	-0.015, 0.013	0.877
Total cholesterol (mg/dL)	-0.002	-0.006, 0.001	0.223
HDL (mg/dL)	0.006	-0.003, 0.015	0.219
Triglycerides (mg/dL)	-0.083	-0.353, 0.186	0.544
HEI	-0.001	-0.009, 0.008	0.855

B, unstandardized regression coefficient; 95%CI, 95% confidence interval; BMI, body mass index; HDL, high-density lipoprotein; HEI, healthy eating index.
